# Complete Genome Sequences of Broad-Host-Range *Pseudomonas aeruginosa* Bacteriophages ΦR18 and ΦS12-1

**DOI:** 10.1128/genomeA.00041-16

**Published:** 2016-05-05

**Authors:** Takaaki Furusawa, Hidetomo Iwano, Hidetoshi Higuchi, Masaru Usui, Fumito Maruyama, Ichiro Nakagawa, Hiroshi Yokota, Yutaka Tamura

**Affiliations:** a Laboratory of Veterinary Biochemistry, School of Veterinary Medicine, Rakuno Gakuen University, Hokkaido, Japan; b Laboratory of Veterinary Hygiene, School of Veterinary Medicine, Rakuno Gakuen University, Hokkaido, Japan; c Laboratory of Food Microbiology and Food Safety, School of Veterinary Medicine, Rakuno Gakuen University, Hokkaido, Japan; d Department of Microbiology, Kyoto University Graduate School of Medicine, Kyoto, Japan

## Abstract

*Pseudomonas aeruginosa* is an important cause of racehorse keratitis. Bacteriophage therapy has the potential to aid in the prevention and treatment of diseases caused by *P. aeruginosa*. We present here the complete genome sequences of two phages, ΦR18 and ΦS12-1, which exhibit infectivity for a broad range of *P. aeruginosa* isolates.

## GENOME ANNOUNCEMENT

*Pseudomonas aeruginosa* is a major causative bacterium of ulcerative keratitis in racehorses (1). This pathogen secretes various proteases, including elastase, which is known to degrade corneal collagen. Therefore, bacterial keratitis caused by *P. aeruginosa* tends to be aggravated (2). Moreover, *P. aeruginosa* and other pathogens are attaining antibiotic resistance throughout the world (http://www.who.int/mediacentre/news/releases/2014/amr-report/en/). To solve the antibiotic resistance problem, phage therapy has attracted the interest of many researchers (3, 4).

Phages ΦR18 (belonging to *Podoviridae*) and ΦS12-1 (belonging to *Myoviridae*), both of which were isolated from sewage treatment plants at Hokkaido in Japan, were purified from their lysate with CsCl density gradient ultracentrifugation, and their DNA were extracted. We submitted the corresponding DNAs to Hokkaido System Science Co., Ltd., for whole-genome sequencing. The samples were sequenced as paired-end reads on the Illumina HiSeq 2500 (Illumina, Inc., San Diego, CA). A total of 1,129,756 and 1,116,928 reads, each with mean lengths of 100 bp, were obtained from ΦR18 and ΦS12-1, respectively. The obtained reads were assembled *de novo* using Velvet (version 1.2.8) (5). Sequences were annotated using the Rapid Annotation using Subsystem Technology (RAST) server (6).

Overwrapped bases were recognized in both sequences. Therefore, we found two DNAs to be circular. The genomic double-stranded DNA of ΦR18 consists of 63,560 bp, with a G+C content of 60.35%. The genome was scanned for coding DNA sequences (CDSs); a total of 86 CDSs were detected. The ΦR18 genome is 97% identical to that of the *P. aeruginosa* phage KPP25 (GenBank accession no. AB910393) (7). ΦR18 has two unique sequences of about 150 bp and 400 bp in length, including CDSs of unknown functional proteins. The ΦS12-1 phage has a genome of 66,257 bp, with 55.58% G+C content; 94 CDSs were detected. The ΦS12-1 genome is 97% identical to that of *P. aeruginosa* phage vB_PaeM_PAO1_Ab27 (GenBank accession no. LN610579) (8). ΦS12-1 has about 3,000 bp of unique sequence, including CDSs for the putative tail protein, putative internal protein, and 4 hypothetical proteins (function is unknown). The endolysins of both the ΦR18 and ΦS12-1 phages have two lysozyme-like domains. Neither of these phages have indicators of lysogenicity or toxic proteins, as detected by the LLNL Virulence Database (9).

In a fundamental aspect, the relationships between genome information and host range variation of these phages might provide insights into mechanisms of host specificity. In addition, information on endolysins, the agents for potential alternatives to antibiotics, is shown in this study and will be very useful for the development of endolysin therapy.

### Nucleotide sequence accession numbers.

The complete genome sequences of ΦR18 and ΦS12-1 have been deposited in the DNA Data Bank of Japan (DDBJ) under accession numbers LC102729 and LC102730, respectively.
